# Innovatives eLearning-Tool für amputierte Menschen in der Ukraine und in Deutschland: Digitale Hilfe zur Selbsthilfe in der sektorenübergreifenden rehabilitativen Versorgung

**DOI:** 10.1007/s00113-025-01543-4

**Published:** 2025-03-07

**Authors:** C. Egen, C. Gutenbrunner, J. Ernst, C. Sturm, J. Schiller

**Affiliations:** 1 Klinik für Rehabilitations- und Sportmedizin, Medizinische Hochschule Hannover, Carl-Neuberg-Str. 1, 30625 Hannover, Deutschland; 2 Klinik für Unfallchirurgie, Medizinische Hochschule Hannover, Hannover, Deutschland

**Keywords:** Obere Extremität, Untere Extremität, Prosthesen, Rehabilitation, Computerassistierte Instruktion, Upper extremity, Lower extremity, Prostheses, Rehabilitation, Computer-assisted instruction

## Abstract

**Hintergrund:**

Nach dem russischen Angriffskrieg sind die Fallzahlen arm- und beinamputierter Menschen in der Ukraine sprunghaft gestiegen und trafen auf ein sich gerade erst im Aufbau befindendes Rehabilitationssystem.

**Ziel der Arbeit:**

Dieser Beitrag beschreibt die Entwicklung und Implementierung eines eLearning Tools, das sich an medizinisches Fachpersonal, Betroffene und Angehörige richtet, vielfältige Informationen zur Rehabilitation nach Amputation von Extremitäten enthält und einen niederschwelligen Zugang bietet. Es soll den Rehabilitationsprozess betroffener Menschen praktisch unterstützen und ist kostenlos in ukrainischer sowie in deutscher Sprache verfügbar.

**Material und Methoden:**

Ein interdisziplinäres Team aus Ärzt*innen, Therapeut*innen, Orthopädietechniker*innen und einem Wissenschaftler gestalteten in einem Konsensusprozess die Struktur und Gliederung des Lernmoduls. Die Inhalte wurden in Kleinteams erarbeitet und nach einem internen inhaltlichen Review durch 2 Teammitglieder sprachlich synchronisiert und auf der Open-Source-Lernplattform Integriertes Lern‑, Informations- und Arbeitskooperations-System (ILIAS) implementiert.

**Ergebnisse und Diskussion:**

Innerhalb von 10 Monaten war das eLearning Tool vollständig entwickelt und implementiert. Über https://digitale-Lehre-mhh.de steht es bilingual und kostenlos zur Verfügung. Es unterscheidet zwischen Rehabilitation nach Arm- oder Beinamputation, anschließend sind 3 zeitlich aufeinanderfolgende Rehabilitationsphasen (postoperative, Interims- und Erhaltungsphase) auswählbar. In der nächsten Ebene sind die konkreten Inhalte abrufbar, die in der Regel aus einem Pathway, erklärenden Texten, praktischen Tipps sowie Abbildungen, Videosequenzen und Audiodateien zur Behandlung und für Selbstübungen bestehen.

## Abstract (en)

**Background:**

Following the Russian war of aggression the number of people in Ukraine with amputated arms and legs rose sharply, overwhelming a fledgling rehabilitation system.

**Aim:**

This article describes the development and implementation of an e‑learning tool aimed at medical professionals, amputees and their relatives, which contains a wide range of information on rehabilitation after limb amputation and is easily accessible. It is designed to provide practical support for the rehabilitation process of affected individuals and is available free of charge in the Ukrainian and German languages.

**Material and methods:**

An interdisciplinary team of doctors, therapists, orthopedic technicians and a scientist developed the structure and outline of the learning module in a consensus process. The content was developed in small teams and, after an internal content review by two team members, linguistically synchronized and implemented on the open-source learning platform Integrated Learning, Information and Work Cooperation System (Integriertes Lern‑, Informations- und Arbeitskooperations-System, ILIAS).

**Results and discussion:**

The e‑learning tool was fully developed and implemented within 10 months. It is available free of charge in two languages at https://digitale-lehre-mhh.de. It distinguishes between rehabilitation after arm or leg amputation, after which three consecutive rehabilitation phases (postacute, intermediate and maintenance phases) can be selected. The next level gives access to the specific content, which consists of a pathway, explanatory texts, images, video sequences and audio files on treatment, self-exercises and practical tips.

Die Auswirkungen des russischen Angriffskriegs auf das ukrainische Gesundheits- und Rehabilitationssystem sind enorm. Die verdreifachte Zahl arm- und/oder beinamputierter Menschen stellt v. a. für die Prothetik/Orthopädietechnik, Physikalische und Rehabilitative Medizin, Ergo‑, Physio- und Psychotherapie eine große Herausforderung dar. Zu deren Bewältigung entwickelte die Medizinische Hochschule Hannover (MMH) in Zusammenarbeit mit weiteren Kooperationspartnern das Lernmodul Rehabilitation after Amputation^Teaching Tool^ (RehAmp^TT^), dessen Entwicklung und Implementierung vorgestellt werden.

## Hintergrund

In Deutschland sind die Fallzahlen von Majoramputation an der unteren Extremität (Operationen- und Prozedurenschlüssel [OPS] 5‑864.0 bis OPS 5‑865.0) seit Erfassung (2005) der entsprechenden Operation anhand des OPS-Katalogs kontinuierlich gesunken. Majoramputationen an der oberen Extremität (OPS 5‑862) kommen äußerst selten vor [1]. Seit dem Ausbruch des Angriffskriegs durch Russland im Februar 2022 steigen die Zahlen in der Ukraine deutlich an. Eine im Februar 2024 offiziell verlautbarte Zahl für die ersten beiden Kriegsjahre belief sich auf über 100.000 Arm- und Beinamputationen seit Kriegsbeginn [2]. Die kriegsbedingten Verletzungen weisen Besonderheiten auf, da häufig aufgrund von Schrapnellen und Granatsplittern auch die obere Extremität betroffen ist bzw. es sich vielfach um Mehrfachamputationen handelt. Ein Drittel der betroffenen Personen ist schätzungsweise überwiegend an der oberen und zwei Drittel sind überwiegend an der unteren Extremität amputiert. Auch liegen häufig weitere Verletzungen z. B. der Augen vor, was eine Rehabilitation entsprechend negativ beeinflusst.

Während der ersten beiden Jahre des Ukrainekriegs erfolgten schätzungsweise über 100.000 Arm- und/oder Beinamputationen

Dem Anstieg dieser Fallzahlen stehen allerdings nur 300 Orthopädietechniker in der Ukraine gegenüber [3]. Gemäß einer *Lancet*-Publikation fehlt es in der Ukraine an ausgebildetem Fachpersonal für die rehabilitative Versorgung der vielen amputierten Menschen – v. a. aber an Fachärzten der Physikalischen und Rehabilitativen Medizin [3]. Ein Mangel besteht auch bei Physio- und Ergotherapeuten – letztere Berufsgruppe gibt es in der Ukraine erst seit ca. 10 Jahren, und die Ausbildung wird seit 2016 auch als Bachelor- und seit 2019 als Master-Studiengang angeboten [4].

In Deutschland beträgt das durchschnittliche Alter beinamputierter Menschen aufgrund der überwiegend vaskulären Ursachen ca. 70 Jahre [1], in der Ukraine sind überwiegend junge Menschen im erwerbsfähigen Alter betroffen. Die Versorgung dieser Altersgruppe mit Prothesen sowie mit rehabilitativen Maßnahmen wird die Ukraine voraussichtlich Jahrzehnte beschäftigen. Und auch nach einem Ende des Krieges wird es durch die vielen verminten Gebiete prognostisch zu weiteren Verletzungen mit Amputationsfolge in der Zivilbevölkerung kommen.

Vor dem Hintergrund des russischen Angriffskriegs auf die Ukraine und seiner Auswirkungen auf das ukrainische Rehabilitationssystem entwickelte die Klinik für Rehabilitations- und Sportmedizin in Kooperation mit der Klinik für Unfallchirurgie der Medizinischen Hochschule Hannover (MHH) und den versorgungserfahrenen, orthopädietechnischen Firmen Brandes & Diesing OHG (Hannover) sowie John + Bamberg GmbH & Co. KG (Hannover) das Lehr- und Lern-Tool Rehabilitation after Amputation^Teaching Tool^ (RehAmp^TT^). Einige Abbildungen und Videosequenzen in dem Tool wurden darüber hinaus von der Fa. Ottobock SE & Co. KGaA (Duderstadt) zur Verfügung gestellt. Gefördert wurde das Vorhaben vom Bundesministerium für Gesundheit (BMG; Förderkennzeichen: 2522IGW007).

## Zielgruppe, Zielstellung und methodisches Vorgehen

Zielgruppen von RehAmp^TT^ stellen sowohl medizinisches Fachpersonal, das bislang wenig bis gar nicht mit der rehabilitativen Versorgung amputierter Menschen konfrontiert war, als auch Betroffene und Angehörige dar. Durch diese heterogenen Zielgruppen sollten eine entsprechend hohe Verbreitung und Nutzung erreicht werden. Ziel des Projekts war es, ein Lehr- und Lern-Tool mit niederschwelligem Zugang, das einen holistischen Ansatz verfolgt und intuitiv von den oben genannten Zielgruppen in der Ukraine und in Deutschland anwendbar ist, zu entwickeln und zu implementieren. Alle Phasen der rehabilitativen Versorgung arm- und beinamputierter Menschen werden grundlegend abgedeckt.

Ein Team aus 13 Personen mit langjähriger Erfahrung im Bereich der Amputationsversorgung war an der Entwicklung und Implementierung des Lernmoduls beteiligt. Zunächst wurden die Struktur und die inhaltliche Ausgestaltung der einzelnen Themen des Tools in einem interdisziplinären Austausch zwischen beteiligten Ärzt*innen, Therapeut*innen, Orthopädietechniker*innen und Wissenschaftler*innen diskutiert und in einem sektorenübergreifenden Konsensusprozess beschlossen. Gemeinsam wurden die Grundstruktur sowie Nutzung grafischer und medialer Darstellungsmöglichkeiten festgelegt. Anschließend erfolgte die inhaltliche Erstellung der Themenbeiträge in den zuvor gebildeten Teams (z. B. Bewegung: Physiotherapie und Fachärzte für Physikalische und Rehabilitative Medizin) gemäß klinischer Praxis. Nach Erstellung der Beiträge erfolgte ein Review durch 2 Experten – einen ärztlichen und einen wissenschaftlichen Mitarbeiter –, die über entsprechende inhaltliche, didaktische und digitale Kompetenzen verfügten. Der Fokus des Reviewprozesses lag auf der inhaltlichen Priorisierung und sprachlichen Synchronisation der Texte sowie deren zielgruppenspezifischer Ausrichtung und Aufbereitung. Um diese Herausforderungen erfolgreich zu bewältigen, war ein enger persönlicher Austausch aller Teammitglieder essenziell. Nachdem die Inhalte von den zwei Gutachtern kontrolliert, ergänzt und sprachlich überarbeitet waren, wurden diese durch einen zertifizierten Dienstleister in die ukrainische Sprache übersetzt.

Zielgruppen von RehAmp^TT^ sind medizinisches Fachpersonal sowie Betroffene und ihre Angehörigen

Zur technischen Umsetzung der Inhalte wurde die Lernplattform Integriertes Lern‑, Informations- und Arbeitskooperations-System (ILIAS) der Bundesarbeitsgemeinschaft digitale Lehre an den Hochschulen für den öffentlichen Dienst in Deutschland genutzt. Die Open-Source-Software ILIAS wird unter der GNU General Public License (GPL) veröffentlicht, sodass sie kostenfrei mit Inhalten versehen und ebenfalls kostenfrei von der Allgemeinheit genutzt werden kann. Die technische Betreuung der bilingualen Digitalisierung der zuvor erarbeiteten Inhalte in ILIAS wurde durch Mitarbeiter*innen des Peter L. Reichertz Institut für Medizinische Informatik der MHH übernommen. Die beiden o.g. Gutachter platzierten und designten in einem letzten Schritt die Inhalte in ILIAS in enger Abstimmung mit den Mitarbeiter*innen des Peter L. Reichertz Instituts.

## Ergebnisse

### Entwicklung und Implementierung

Der Umfang des zu vermittelnden Faktenwissens ist aufgrund der sektorenübergreifenden Behandlungsstrategien der Rehabilitation von arm- und/oder beinamputierten Menschen enorm, und die Fokussierung auf die wesentlichen Inhalte stellte eine Herausforderung dar. Nichtsdestotrotz konnten innerhalb des vorgesehenen Zeitrahmens von 8 Monaten (vom 01.01.2023 bis 31.08.2023) alle Inhalte fertiggestellt werden. Nach weiteren 2 Monaten (bis 31.10.2023) waren alle Inhalte ins Ukrainische übersetzt sowie in ILIAS bilingual und anwenderfreundlich implementiert.

### Inhalte

**Abb. 1:**
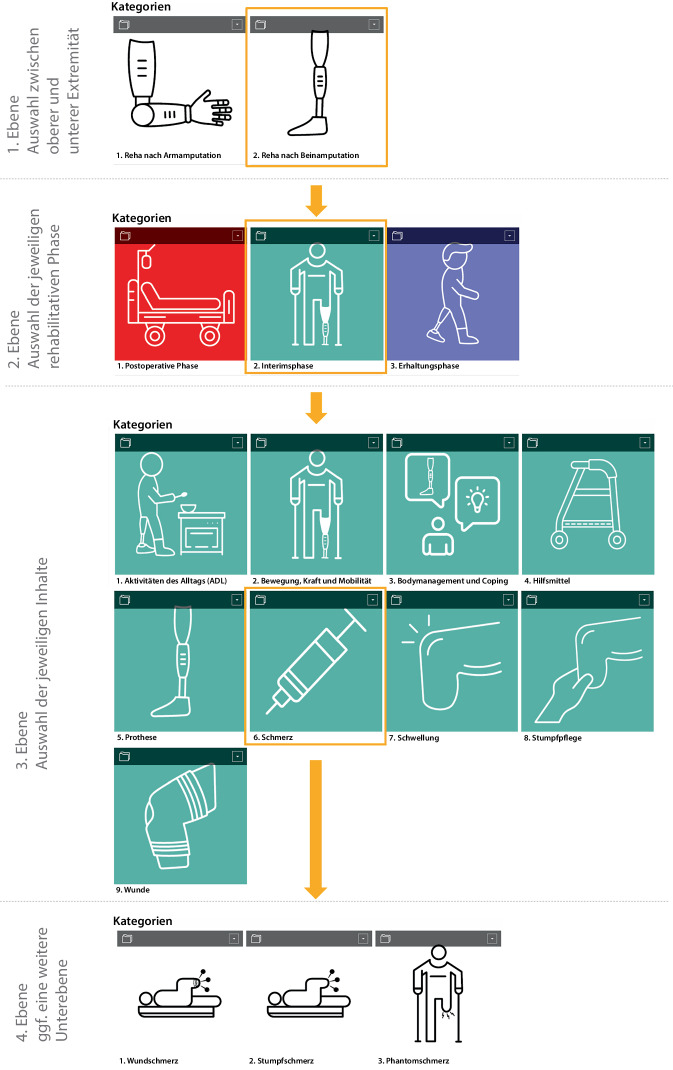
Exemplarische Darstellung der grafischen Umsetzung

Die konkreten Inhalte bestehen aus einem Pathway, erklärenden Texten, praktischen Tipps sowie Abbildungen, Videosequenzen und Audiodateien zur Behandlung und für Selbstübungen. Die einzelnen Verlaufsphasen sowie die 9 Schwerpunktthemen wurden farblich und durch intuitiv verständliche Icons voneinander abgegrenzt (Abb. 1). Die verwendete Datenmenge der Bilder und Videos wurde bewusst limitiert, damit eine Benutzung auch bei schlechten Internetverbindungen möglich ist. Das Lernmodul kann sowohl über ein Mobiltelefon als auch einen PC benutzt werden.

So entstanden 78 Internetseiten mit unterschiedlichem inhaltlichem Umfang. Der reine Text ohne Pathways (Behandlungspfade), Grafiken, Bilder und Videos beläuft sich auf 85 MS Word-Seiten (Schriftgröße 12) ohne Berücksichtigung von Duplikaten. Manche Inhalte sind in 2 oder gar allen 3 Phasen identisch, wie beispielsweise die Inhalte bei den Themen „Schmerz“ und „Hilfsmittel“, sodass der Gesamtumfang deutlich größer ist. Unter https://digitale-lehre-mhh.de kann auf RehAmp^TT^ kostenlos zugegriffen werden.

#### Phasen der rehabilitativen Versorgung

Das Tool führt schrittweise durch die Phasen der rehabilitativen Versorgung und stellt die Behandlungstechniken im zeitlichen Verlauf pragmatisch dar. Grundsätzlich wird zunächst zwischen „oberer“ und „unterer Extremität“ unterschieden. Auf der nächsten Ebene sind 3 zeitlich aufeinanderfolgende und farblich klar voneinander abgegrenzte Behandlungsphasen („postoperative Phase“, „Interimsphase“ und „Erhaltungsphase“) auswählbar.

Alle Phasen der rehabilitativen Versorgung arm- und beinamputierter Menschen werden abgedeckt

In der *postoperativen Phase* im Akutkrankenhaus stehen sowohl bei arm- als auch beinamputierten Menschen die Wundheilung, die Ödemreduktion, die Kontrakturprophylaxe, die Frühmobilisation, die Stumpfkonsolidierung und -formung sowie die Schmerztherapie im Vordergrund der Behandlung. Ziele der *Interimsphase* sind die vollständige Wundheilung, eine Konsolidierung der Stumpfverhältnisse, die Fertigstellung der Interimsprothese und das erste Gangtraining mit der Interimsprothese. Danach würde sich in der Mehrzahl der Fälle die medizinische Rehabilitation anschließen; deren Inhalte sind idealtypischerweise auf die individuellen Bedürfnisse der Betroffenen ausgerichtet. In der *Erhaltungsphase* stehen Selbstübungen im Vordergrund, die die zuvor erworbenen Fähigkeiten und Kompetenzen vertiefen und in den Alltag umsetzen.

#### Schwerpunktthemen

Nach Auswahl der Behandlungsphase wird dem Nutzer eine Auswahl von folgenden 9 Themenbereichen, die sich anhand von intuitiv verständlichen Piktogrammen unterscheiden, offeriert:

##### Aktivitäten des täglichen Lebens.

Nach einer Amputation ergeben sich für Betroffene vielfältige Einschränkungen in den Aktivitäten des täglichen Lebens. Neben dem Training mit Prothesen und verschiedenen Hilfsmitteln müssen Verhaltensänderungen eingeübt werden, um eine möglichst große Selbstständigkeit zu erreichen. Das Lernmodul stellt eine Möglichkeit des Abfragens von persönlichen Ressourcen zur Therapieplanung zur Verfügung, informiert über die Erfassung des individuellen Tagesprofils und gibt praktische Hinweise und Tipps für Alltagsaktivitäten (Duschen, Ankleiden, Spazierengehen, Treppensteigen etc.) mit und ohne Hinzunahme von Hilfsmitteln. Auch über Anpassungsmöglichkeiten der Wohnumgebung und des Arbeitsplatzes wird informiert.

##### Bewegung, Kraft und Mobilität.

Dieser Themenbereich nimmt eine zentrale Stellung im Modul ein. In der postoperativen Phase stehen Pneumonieprophylaxe, kontraktur- und thrombosevorbeugende Maßnahmen sowie die rasche Ödemreduktion und -resorption im Vordergrund der Therapie. Über dementsprechende Eigenübungen wird im Lern-Tool informiert. Darüber hinaus werden praktische Übungen zu Mobilisation, Dehnung und Kraft sowie zu Rumpfstabilität und Feinmotorik durch Videos und Bilder angeleitet. In den späteren Phasen werden Übungen und Therapien mit Prothesen und zur Prothesennutzung aufgezeigt.

##### Bodymanagement und Coping.

In diesem Bereich wird über die verschiedenen Phasen der psychischen Verarbeitung einer Amputation informiert. Es werden Bewältigungsstrategien zum Umgang mit der neuen Lebenssituation bzw. der körperlichen Veränderung aufgezeigt sowie Hinweise zur Vermeidung maladaptiver Strategien gegeben.

##### Hilfsmittel.

Die wesentlichen Hilfsmittel und ihre Einsatzmöglichkeiten – differenziert nach Arm- oder Beinamputation – werden dargestellt. Hilfsmittel sind für amputierte Menschen ein essenzieller Bestandteil zur Bewältigung des Alltags und zur Kompensation der Beeinträchtigungen.

##### Prothese.

Die Prothese ersetzt im optimalen Fall das amputierte Körperteil, übernimmt wichtige Funktionen und stellt das äußere Erscheinungsbild wieder her. Eine individuelle angepasste und passgenaue prothetische Versorgung soll eine Integration in das Körperschema ermöglichen, um normale Bewegungsabläufe und die Teilhabe zu ermöglichen. Die verschiedenen Prothesentypen, ihr Aufbau und individuell an die Bedürfnisse angepasste Prothesenkomponenten (Passteile) werden informativ dargestellt.

##### Schmerztherapie.

Eine konsequente Schmerztherapie, die sich aus medikamentösen, physikalisch-medizinischen und schmerzpsychologischen Maßnahmen zusammensetzt, sollte zu jedem Zeitpunkt verfügbar sein, um einerseits eine Chronifizierung und Phantomschmerzen zu vermeiden und andererseits die Mobilisierung zum frühestmöglichen Zeitpunkt zu ermöglichen. Es wird auf phasenspezifische und schmerzspezifische (Wund‑, Stumpf‑, Phantomschmerz, neuropathischer Schmerz) analgetische Medikationen eingegangen, und konkrete Beispielmedikationen werden benannt. Zusätzlich wird über die Durchführung von Spiegeltherapie und optionalen Verfahren wie thermotherapeutischen Maßnahmen, CO_2_-Therapie, sensorisch-perzeptivem Training und Elektrotherapie informiert. Entspannungstechniken werden den Betroffenen mithilfe von Audioinhalten angeboten.

##### Schwellung des Stumpfes.

Nach der Amputation besteht ein posttraumatisches sekundäres lokales Ödem mit ausgeprägter Schwellung des Stumpfes, dass mithilfe entstauender Maßnahmen reduziert werden sollte, um die Wundheilung zu beschleunigen und eine zeitnahe Anpassung der Prothese zu ermöglichen. Hierzu werden die Maßnahmen der komplexen physikalischen Entstauungstherapie, die neben der manuellen Lymphdrainage eine Kompressionsbehandlung sowie Bewegungs- und Atemtherapie beinhaltet, und die Lagerung der betroffenen Extremität erklärt. Auch die zirkuläre Anlage von Kompressionswickeln wird mit Bildmaterial dargestellt sowie auf die Gefahren und typischen Fehler hingewiesen. Zusätzlich wird über die Materialien von Linern, Kompressionsbestrumpfung und ihre korrekte Anlage informiert.

##### Stumpfpflege.

Nach Entfernung des Nahtmaterials und Abschluss der oberflächlichen Wundheilung ist dem Betroffenen eine korrekte Stumpfpflege zu vermitteln, um Komplikationen wie Druckläsionen und Berührungsüberempfindlichkeit zu vermeiden sowie die Region auf die zukünftigen Belastungen im Rahmen der Prothesennutzung vorzubereiten. Es wird über Desensibilisierungsmaßnahmen, Optimierung der Durchblutung, Hygienemaßnahmen und oberflächliche Hautpflege unter Nutzung einfacher Hilfsmittel (u. a. weiche Bürste, Tuch, Tennis‑/Igelball, Spiegel) mithilfe eindeutiger visueller Darstellungen informiert, sodass die Betroffenen konkrete Handlungsanweisungen erhalten, um ihren Stumpf ohne personelle Unterstützung zu pflegen.

##### Wundheilung.

Die Wundheilung spielt besonders in der Anfangsphase eine enorm wichtige Rolle, da die Anpassung einer Prothese und der hiermit verbundene Mobilitätsgewinn prioritäre Ziele für die Rehabilitand*innen darstellen. Phasenabhängig wird im Lernmodul auf entsprechende amputationsspezifische Komplikationen der Wundversorgung eingegangen. Handlungsmaßnahmen werden dezidiert durch bebilderte Beispiele erläutert. In der frühen Phase nach einer Amputation wird neben Infektionen, Hämatomen, Wunddehiszenzen, Stumpfsekretion und Nekrosen über konkrete Beispiele und die entsprechenden Behandlungsstrategien informiert. In der Erhaltungsphase wird auf Narbenbehandlung, verruköse Hyperplasie, Abszesse und Druckläsionen sowie ihre Folgen eingegangen. Auch die Themen Kontaktdermatitis, Haarfollikulitis und Neurome stehen in dieser Behandlungsphase im Fokus.

## Diskussion

Die Entwicklung von RehAmp^TT^ entstand als Reaktion auf den akut angestiegenen Bedarf an rehabilitativer Versorgung für Menschen nach Gliedmaßenamputationen in der Ukraine, der im bestehenden Versorgungssystem nicht gedeckt werden konnte. Dies bezieht sich sowohl auf die frühe postakute als auch die Interims- und die Langzeitversorgung. Neben dem Mangel an Rehabilitationseinrichtungen war auch ein Mangel an spezialisierten Fachkräften festzustellen, der in besonderem Maße die Orthopädietechnik betraf. Hieraus leitete sich der Ansatz des Lernmoduls ab, das sich nicht nur an Fachkräfte, sondern in besonderem Maß auch an nichtspezialisiertes medizinisches Fachpersonal, aber auch an die Betroffenen und ihre Familien richtet. Aus diesem Grund war es unabdingbar, die Informationen in ukrainischer Sprache und mithilfe leicht verständlicher Grafiken zu übermitteln. Auf längere erläuternde Texte wurde bewusst verzichtet.

Obwohl das Tool unabhängig von rehabilitationsfachlicher Expertise anwendbar ist und inhaltlich prinzipiell der klinischen Praxis in Deutschland entspricht, versteht es sich als Ergänzung und nicht als Ersatz fachspezifischer Rehabilitationsprogramme. Vielmehr solle es zum (graduellen) Ausgleich von Versorgungslücken beitragen und kann auch bei nicht mehr bestehenden Versorgungsdefiziten als Ergänzung und zur Verstetigung von Rehabilitationsprogrammen dienen.

Sogenannte Tele-Rehabilitationsprogramme wurden in den letzten Jahren auch für andere Indikationen entwickelt. Ein Treiber für diese Entwicklung war die letzten Jahre v. a. die COVID-19-Pandemie, in der viele Rehabilitationsleistungen nicht mehr – oder nur unter erschwerten Bedingungen – hands-on durchgeführt werden konnten. Dabei es handelt sich um App-gestützte, video- und onlinebasierte Programme [5–7]. Für einige dieser Programme liegen mittlerweile erste Ergebnisse der wissenschaftlichen Evaluation vor [8–10]; diese sind naturgemäß noch sehr uneinheitlich.

Das Lern-Tool versteht sich als Ergänzung zu fachspezifischen Rehabilitationsprogrammen

Für das in diesem Beitrag vorgestellte Lernmodul wäre es selbstverständlich wünschenswert, eine Evaluation zur Nutzung, zur Verständlichkeit und zur Wirksamkeit durchzuführen. Dies konnte aus organisatorischen, finanziellen und kapazitativen Gründen in der Hauptzielgruppe bisher nicht durchgeführt werden. Obwohl das Tool in erster Linie für die spezielle Situation in der Ukraine entwickelt worden ist, ist in Betracht zu ziehen, ob eine Evaluation auch im deutschen Rehabilitationssystem machbar und aussagekräftig wäre.

Darüber hinaus sollte RehAmp^TT^ auch für andere Länder verfügbar gemacht werden, insbesondere in Ländern, die über keine oder nur eine geringe Anzahl an spezialisierten Rehabilitationseinrichtungen für Menschen nach Gliedmaßenamputationen verfügen. Hierfür wären aber Übersetzungen in die jeweiligen Landessprachen notwendig. Ein weiterer Anwendungsbereich könnte die Verwendung in der medizinischen Aus- und Weiterbildung sein, wobei ausdrücklich Angehörige der Gesundheitsfachberufe und Pflegefachkräfte eingeschlossen werden könnten.

RehAmp^TT^ ist seit dem 01.11.2023 online und wurde über verschiedene Netzwerke verbreitet. Bis zum 31.12.2024 wurden ca. 3000 Lesezugriffe, die allerdings nicht länderspezifisch auswertbar sind, verzeichnet. Das Lernmodul wurde über eine Pressemitteilung [11], verschiedene Artikel bzw. Interviews [11, 12], entsprechende Netzwerke und Kongressbeiträge beworben [13]. Ob es in der Ukraine tatsächlich Anwendung findet, kann an dieser Stelle, wie bereits erwähnt, aufgrund der fehlenden Evaluation, nicht final eruiert werden.

## Fazit für die Praxis

- In der klinischen Routine wird das Rehabilitation after Amputation^Teaching Tool^ (RehAmp^TT^) bereits erfolgreich in der Medizinischen Hochschule Hannover (MHH) eingesetzt, um die einzelnen Schritte und den Rehabilitationsverlauf zu unterstützten.
- Neben der Wissensvermittlung an Betroffene, deren Angehörige und in den Behandlungsprozess involvierte Behandler ist auch die Nutzung in der ärztlichen und therapeutischen Weiterbildung sowie in der studentischen Lehre eine sinnvolle Ergänzung.
- Allen Anwender*innen wird empfohlen, das Lernmodul im Alltag bzw. in der Praxis anzuwenden und den Autoren zurückzuspiegeln, ob es eine sinnvolle Ergänzung zur Rehabilitation darstellt. Gleichzeitig sind die Autoren für Rückmeldungen zur Weiterentwicklung offen und dankbar.
